# ‘Bad for the Health of the Body, Worse for the Health of the Mind’: Female Responses to Imprisonment in England, 1853–1869

**DOI:** 10.1093/shm/hkz066

**Published:** 2020-01-18

**Authors:** Rachel Bennett

**Affiliations:** Centre for the History of Medicine, University of Warwick, Coventry, CV4 7AL, UK

**Keywords:** prison, women, health, resistance, Brixton

## Abstract

Upon committal to one of the newly established female convict prisons in the mid-nineteenth century, women entered a system intended to regulate them in body and in mind for the ends of reform. This article interrogates how women’s health needs were identified and contested by the prison officials and doctors tasked with their custody and care. It highlights the importance of broader temporal gender beliefs in dictating their treatment in this carceral space and explores how the women themselves exercised agency over the terms of their imprisonment. In addition, it reveals the previously underexplored transference of women between the institutions that made up the female convict estate that was prompted by concerns about the impact of a rigorous prison system on their physical and mental health.

In 1853, the gates of Brixton convict prison were opened for the reception of its first female inmates. One of several convict prisons established in the mid-nineteenth century, Brixton was unique in that it was designated exclusively for women and would remain so until 1869. When reflecting upon his early tenure as the prison’s Medical Officer in 1856, James Rendle remarked that ‘the collecting of so large a number of female prisoners in a prison expressly prepared for women are circumstances altogether new in this country’. He continued, ‘a system of management is unknown, and experience is wanting’.^1^ Using Brixton’s opening and eventual closure as a chronological frame, this article will demonstrate that, although it was intended to be the model prison for women, the regime within required constant adaptation due to considerations about prisoner health. Thus, while Rendle’s remarks reflect the difficulties he faced in his own position, they also illuminate the much broader complexities faced by the prison authorities when managing discipline and health within the new female convict estate, especially as many women were found to be in poor physical and/or mental health upon reception.

Although women were considered to be a distinct category of convict, the system implemented within the newly established female estate was largely a modified version of that designed with male criminals in mind. McConville briefly noted the ‘considerable organisation, staffing and disciplinary problems’ caused by this.^2^ However, subsequent studies have furthered the analysis with Zedner and Priestley, respectively, arguing that, as women consistently accounted for a lesser proportion of the total prison population, their disciplinary needs were an afterthought, or they were treated as ‘rather difficult men’.^3^ Davie later expanded this discussion to explore how female inmates were seen as ‘difficult’ but not necessarily as ‘difficult men’. Instead, their impulsive natures were blamed for creating an ‘unexpected problem of discipline’.^4^ This article examines how this ‘problem’ was further complicated when considered alongside the equally unprepared for question of managing female health behind bars.

Dobash, Dobash and Gutteridge explored how class and gender assumptions shaped the way prisons for women were organised in the nineteenth and twentieth centuries.^5^ Zedner’s study of the Victorian period furthered this by charting how shifting explanations for female criminality impacted upon efforts to make prisons more female-oriented in their approach to discipline.^6^ This article builds upon the valuable insights into the regulation of behaviour offered by these studies with an exploration of the provisions in place to care for the distinct health needs of female inmates alongside the specific disciplinary problems they were believed to pose. It advances our understanding of how those tasked with the carceral containment of women, including the medical officer, negotiated this balancing act.

By virtue of their office, the prison medical officer was a constable, as well as a doctor. They determined a prisoner’s fitness for work and subjugation to the prison regime, including certifying them as fit or unfit for labour, dietary punishment and restraint. Wiener labelled this decision-making process a form of ‘moral categorisation’ that involved the interpretation of behaviour, as well as the identification of ill health.^7^ Sim and Priestley, respectively, critiqued what they argued to be the fundamentally disciplinary task of the prison doctor that placed them more on the side of the state than the sick.^8^ Focusing upon female convict prisons between 1853 and 1869, this study questions how the distinct medical, emotional and disciplinary needs of women were understood and rationalised by those tasked with their custody, notably the medical officer. It uncovers the previously underexplored transfer of women between the different prisons that made up the female convict estate, and argues that this movement was not solely based upon progression through the disciplinary classes earned by good behaviour, as initially intended in 1853. Rather it was influenced by debates about health, behaviour and suitability for the prison regime.

To gain an insight into the provision for and management of female health in prison, this article draws from a range of materials, including accounts written by prisoners and staff, as well as contemporary discourse deliberating female criminality. An extensive reading of Prison Commission and Home Office records and the Reports of the Directors of Convict Prisons provides valuable information on the number of women committed and released each year, detailing any medical treatment they received and recording the punishments meted out for infractions of the prison rules. We must acknowledge that within official records, the prisoner’s voice was often absent or mediated by those recording the information. However, a reading of their content enables an assessment of the ways in which prison officials identified, categorised and rationalised health and disciplinary needs and, crucially, how these assessments impacted upon the punitive and medical treatment of women in prison.

## Establishing the Female Convict System 

The Penal Servitude Act 1853 replaced sentences of transportation, which had ceased to be a punishment option for women in 1852 when Van Diemen’s Land announced that it would no longer accept female transportees, with those of penal servitude. Initially, the minimum length of a sentence was 4 years, but following the passing of the Penal Servitude Act 1857, sentences ranged between 3 years and life. They were served in a government-controlled convict prison where prisoners and prison staff lived and worked as part of heavily regulated regimes. The current study does not have the scope to delve too deeply into the long-term debates underpinning the creation of the convict prison estate and the contested rationale of its early disciplinary regimes. There is extensive scholarship devoted to such analyses, notably Volume I of McConville’s seminal study *A History of English Prison Administration*. However, a brief introduction into the establishment of the female convict estate between 1853 and 1869 is useful here.

The Surrey House of Correction was purchased and adapted by the government in 1852 to create Brixton, Britain’s first female convict prison. It was designed by the Chairman of the Directors of Convict Prisons, Sir Joshua Jebb, who was often consulted by the Home Office in matters of prison construction due to his prior positions in the Royal Engineers and as Inspector-General of Military Prisons. At the outset of this new penal experiment in November 1853, 75 prisoners were transferred in from Millbank, and by June 1854, there were over 550 prisoners incarcerated in Brixton. However, overcrowding prompted the decision to reallocate one of Millbank’s pentagons to women in February 1855. It was decided that, henceforth, women would undergo the probation stage of discipline and the third class in Millbank before progressing on to Brixton to complete the second– and first-class stages. Prisoners would be lectured on the nature of classification and the means of progression by showing industriousness and maintaining good behaviour. Each promotion involved a gradual increase in association with other prisoners, as they moved from cellular isolation, which entailed very limited contact with prison staff, and especially with other prisoners, to silent association, where they could work alongside each other but were not permitted to communicate in any way. They would then progress to a more advanced state of association and communication.^9^ Fulham Refuge was opened in 1856 to receive the women who had shown impeccable behaviour to provide them with industrial training in the final months of their sentence with the aim of helping them to find respectable employment upon release, usually in domestic service.^10^

The female convict estate in this period became a system wherein women were moved between prisons, but it was not the smooth system initially envisaged by Jebb based solely upon progression due to compliance with the prison rules. Instead, the regime had to be negotiated and adapted by prison officials due to concerns about prisoners’ health. For example, some women were deemed unfit for the discipline in Millbank and removed to Brixton despite their conduct not warranting such progression. In contrast, women who had earned their progression to Fulham Refuge due to good behaviour were deemed unfit for a place there, often due to age or debility, and were thus detained in Brixton until the expiration of their sentence.

The prison doctor’s statutory duty to distinguish between those fit and unfit for prison labour and punishment was a difficult one and, Watson argued, facilitated the production of knowledge and debates about categories of mental behaviour that were unique to the prison setting.^11^ Davie added that it was both practical and crucial for prison doctors to establish objective criteria to reflect upon the distinct nature and extent of physical and mental disabilities among prison inmates to not only decide upon their fitness to undergo the full rigours of the regime but to also pre-empt any challenges to their diagnoses from other quarters of the prison hierarchy.^12^ When reporting upon his first year as Brixton’s Medical Officer, Rendle, who had previously been Millbank’s resident surgeon, stated his belief that ‘women as a body do not bear imprisonment so well as the male prisoners; they get anxious, restless, more irritable in temper, and are more readily excited’.^13^ Specific peculiarities he encountered were cases of ‘breaking out’.

Several reports detail instances where women had destroyed their cells, smashed the windows, tore their clothes to pieces, were shouting and singing uncontrollably and threatened to harm themselves and others. These instances were variously described as being prompted by temporary fits of insanity and feeble mindedness but were also labelled as acts of insubordination intended to circumvent the prison rules. Although some of the women were believed to be unresponsive to any punitive or reformatory treatment, they were repeatedly placed in isolation on a punishment diet to prevent further outbursts. Descriptions of this type of disruptive and potentially violent conduct were not confined to female prisons and have been identified within studies of the treatment of women in asylums in the second half of the nineteenth century.^14^ However, the term ‘breaking out’ was coined in the early 1850s by prison doctors and other officials to specifically describe this type of behaviour in female convicts. This categorisation is crucial to our understanding of not only how certain cases were explained but also how they were responded to and treated.

Prior to the mid-nineteenth century, prison sentences were short or acted as temporary preludes to other punishments such as the death sentence or transportation. However, sentences of penal servitude were for a minimum of 3 years and were intended to reform convicts before their release back into society. This process of reformation began with prisoners being placed in separate confinement. The separate system was based on the principle that inmates would have time for individual reflection and would be incarcerated in isolation, with no contact with fellow prisoners, minimal contact with prison officials and only very limited time out of their cells for chapel and exercise. On the basis of the model set out in Philadelphia’s Eastern State Penitentiary, it was first introduced to Britain in Pentonville Prison, for male convicts, in 1842. Cox and Marland demonstrated that, while the system had many detractors from its inception due to its association with cases of mental breakdown, its supporters pointed to this extreme form of separation as an indispensable step along the path to true reform.^15^ Despite ardent criticisms, the separate system was implemented, though modified, in several prisons in the mid-nineteenth century and sustained until the early twentieth century.

The separate system was established in female convict prisons but from the outset prompted debate about the ability of women to withstand the full rigours of separation. Inspector of Prisons, John George Perry, argued in 1850 that the more sedentary habits of women on the outside better equipped them to withstand a restriction of mobility in prison.^16^ Women were also considered to be naturally more sociable, and thus, they would feel more greatly the deprivation of conversation, which was posited as potentially harmful but also as a useful disciplinary tool. For example, in 1851, officials in Hull Gaol were questioned about the effects of the separate system upon the prisoners. Mrs Silvester, the matron in charge of the small number of women, provided a robust approval when she stated, ‘I am fully convinced that nothing but the separate system will tend to prevent crime for at present they are not afraid of returning to gaol as they have sufficient food and congenial society’.^17^ Silvester’s statement encapsulates a criticism that was levelled at the prisons of the past during debates about the adoption of the separate system, namely the moral and disciplinary dangers of incarcerating prisoners in association.

In 1853, the convict prison Directorate issued instructions that women would spend 4 months in separate confinement as opposed to the 9 months specified for male convicts.^18^ Following the reallocation of a pentagon at Millbank to women in 1855, it was decided that they would undergo this part of their sentence there before being considered for promotion to the third class and eventual removal to Brixton. Although the probation stage continued to be for 4 months, in practice, some women in Millbank’s third class were held in separate confinement for longer due to their behaviour but also because of a lack of available accommodation.^19^ Additional modifications included second– and first-class prisoners in Brixton being allowed to do their needlework at the doors to their cells instead of inside them. From October 1855, they were allowed to exercise in pairs for an hour in the morning and an hour in the afternoon.^20^ Following their visit to Brixton, journalists Henry Mayhew and John Binny commented upon the curious sight of 200 women pacing around the exercise yard in pairs, ‘chattering as they go like a large school’.^21^ Alongside recognitions that women were unsuited to the full rigours of separation were claims that a lack of association was a potential cause of ‘breaking out’.

Florence Maybrick, who spent 15 years in prison for the murder of her husband, recalled in her memoir that in separate confinement, ‘all individuality, all friendship, all things that make human beings attractive to one another are absent’. She described how women would shriek loudly, tear their clothes and smash their cell windows when kept in such a condition.^22^ Similarly, Susan Willis Fletcher, who had been imprisoned in Westminster Prison in the early 1880s, described spending 23 hours a day in a tomb-like cell as an experience that was, ‘bad for the health of the body, worse for the health of the mind’.^23^ However, a report made by Rendle in 1854 illuminated the contradictions in beliefs about the effects of separate confinement upon women. He claimed the system would not prove injurious to prisoners in mind or body as they would have contact with the matron and the chaplain, have exercise and attend chapel. Yet he ordered the removal of a woman from the separate cells to be placed in association as she was restless and constantly talking to herself.^24^ Similarly, in 1858, he found that poor conduct was often attributable to a need for association. Brixton already had three large cells that each accommodated up to three women who required this greater association. He recommended building four more.^25^

Following their period in separate confinement, male convicts were sent to public works prisons, including Dartmoor and Portland to undertake forms of outdoor labour.^26^ While demanding, this physical work was believed to be advantageous due to the change in environment it offered. However, outdoor labour was deemed unsuitable for female convicts. Instead, their prison labour predominantly consisted of needlework, making clothes for male prisons and working in the prison’s bakery and laundry; the latter offered some degree of physical exertion but was still considered to be monotonous. Prison officers and doctors repeatedly complained to the prison Directorate about the lack of outdoor labour options for women and the dangers posed to their health by remaining in the same conditions for the duration of their sentence. In 1855, John Henry Moran, Brixton’s Chaplain, warned, ‘women cannot, with safety to mind and body, be subjected to such lengthened periods of imprisonment’. He blamed the sedentary nature of the prison regime for their irritability and reckless conduct, adding that it would be, ‘utterly impossible to retain them for the whole period of their sentence unless the prison is turned into a large hospital or a lunatic asylum’.^27^

Arthur Griffiths, the Deputy Governor of Millbank between 1872 and 1874, bemoaned that some of the women had maintained ‘an unbroken warfare with authority’. He added, ‘it is often difficult to draw the line between madness and outrageous conduct’.^28^ This line was constantly in flux and subject to extensive redrawing by prison doctors when deciding upon the appropriate treatment of inmates. ‘Breaking out’ was often labelled as an example of women feigning weakness of mind to prompt their removal to the prison infirmary to gain greater association with other prisoners and a more substantial diet. This suspicion was not confined to women’s prisons. Cox and Marland found that Pentonville officials were vigilant in their efforts to detect the feigning of insanity and used it as evidence of a prisoner’s intrinsic weakness and incorrigibility to refute claims that the separate system had caused mental breakdown.^29^ Prison doctors increasingly regarded themselves as experts in distinguishing between malingering and mental deficiency due to their extensive and daily observation of prisoners.^30^ However, arrival at reliable and consistent diagnoses remained problematic and, Davie demonstrates, decisions about whether prisoners were fit for labour and discipline sometimes placed prison doctors at odds with other members of the prison hierarchy.^31^

Shepherd argued that feigning insanity was rarely a prisoner’s first way of fighting against the regime but that it was resorted to when other efforts at resistance had failed.^32^ Dobash *et al.* labelled women’s shouting and singing as ‘a common form of protest and self-expression’.^33^ ‘Breaking out’ and other destructive behaviours among female convicts were also believed to have been driven by a desire for some form of association or to get ‘a little variety to their life’.^34^ It was suspected that some women showed evident determination to be sent to the punishment cells where they could communicate with each other more easily. However, Sim argued that whether these destructive acts were underpinned by feigning or rebellion, the bodies of those committing them became the prime focus of the authorities’ debilitative efforts, and thus they ‘forfeited further [their] right to be treated with dignity’.^35^ Recalcitrant bodies were physically restrained using handcuffs, strait jackets and the canvas dress, but they were also placed in isolation to prevent their contagion of other prisoners. There were initially 12 punishment cells in Brixton for this purpose, but in November 1857, an additional 18 were built to separate refractory women as much as possible from the rest of the prison.^36^ Similarly, in an attempt to combat the desire to ‘show off’ among Millbank’s refractory women, a ‘dumb cell’ was created for their containment with mattress-type fixtures placed on the walls and the cell door to prevent the emission of sound.^37^

In this environment of strict regulation, the desire to exercise agency over their imprisonment caused some women to ‘break out’. In 1860, Maria Copes was undergoing the probation stage of discipline in Millbank, having been moved back from Brixton due to her behaviour. She continually broke her cell furniture, and it would take several officers to move her into a dark cell where she would leap from side to side and bash her head against the walls. When it became apparent that she was too violent for the dark cell, she was placed in a canvas jacket, but she managed to rip it up as, according to William Guy, Millbank’s Medical Officer, she had the strength of a man. When handcuffs were used to restrain her, she bit her wrists violently. These incidents, described as ‘paroxysms of passion’, lasted for days, but she was believed to be insensible to the pain she inflicted upon herself. She was eventually confined to a padded cell but used her teeth to tear at the walls. She stated that she would not behave well until she was placed in association but when accommodated in a cell with other women, she had assaulted them. She also made threats against officers and several attempts on her own life, although Guy did not consider them to have been serious attempts.^38^

In November, Captain O'Brien, the Director responsible for the female convict estate, visited the prison. He found Maria handcuffed to the end of her iron bedstead with her ankles secured by a chain. He described being ‘horrified’ by the sight but added that an hour after she was unshackled, she began to bang her head against the wall of the exercise yard and again had to be restrained.^39^ Guy eventually called in Dr Forbes Winslow, a psychiatrist who worked outside of the prison system who was a leading authority in lunacy and its treatment, for a second opinion.^40^ They concurred that Maria was not of unsound mind. Guy used her case to argue that suicide attempts and self-harm could be ‘quite compatible with a sound state of mind’.^41^ However, O’Brien reported to Jebb that he was not convinced of her sanity and worried that her case could be held up to public censure. Therefore, he recommended that Maria be removed to Brixton, where she had stated that she wanted to be.^42^ She was among 21 prisoners moved to Brixton during the year on medical grounds. Her removal was described as being for ‘eccentricity’.^43^ Maria was later released at the end of her sentence.

Maria’s case highlights the complexity of distinguishing between women being deliberately impervious and being immune to punitive efforts at correction, but also the agency the women themselves sought over their fate. Despite the evident concern that she may harm herself through her actions, she was treated as a refractory, rather than a mentally ill, prisoner. Although O’Brien acknowledged that she was unfit for a continuation of the discipline at Millbank, despite Guy’s opinion that she was not of unsound mind, her removal to Brixton was more about disciplinary management than medical treatment. In this sense, Maria’s case provides some reinforcement to the argument recently made by Cox and Marland in relation to male convicts, namely that the management of mental illness in prisons in this period was often aimed at ‘mitigating harm to the institution rather than relieving the prisoner patient’.^44^

In her 1864 work *Our Convicts*, Mary Carpenter labelled instances of women smashing their cells and tearing their clothes as ‘unmistakeable signs of extraordinary mismanagement and a want of control’.^45^ In this period, ideal femininity was intrinsically bound up in docility and restraint from any display of strong emotions.^46^ However, alongside this, women were believed to be more susceptible to irritability and emotional outbursts.^47^ Poor behaviour, including shouting, swearing and destroying prison property, was blamed upon female nature but was also lamented as distinctly ‘unfeminine’ by prison officials and thus requiring especial censure. Prisons for women, and the females who staffed them, were believed to play a key part in the pursuit of morally reclaiming these women.

Emma Martin, Brixton’s Lady Superintendent, commented that it was necessary for female officers to provide a good moral example to the women in their care.^48^ However, Zedner and Dobash *et al.*, respectively, argued that this also prompted greater intolerance for infractions of the prison rules among female inmates.^49^ Forsythe subsequently countered that the difference in treatment based upon sex was less pronounced than Zedner suggested and refuted the assertion made by Dobash *et al.* that female prisoners were punished with greater severity and frequency than their male counterparts. However, he acknowledged that Brixton’s regime was largely a ‘(mal)adaptation of prison regimes for men’ that was informed by male beliefs about female respectability.^50^ These beliefs, and the emphasis placed upon the moral role of women’s prisons and their staff, impacted not only upon the discipline of the institution but also had the potential to shape discussions about female prisoner health.

When he entered Coldbath Fields Prison in the early 1850s to take up the position of Governor, George Chesterton recalled the women standing meekly before him in perfect silence. However, he continued, this was, ‘calculated to lull me into the belief that there stood arrayed before me was the very concentration of gentleness and tractability’. After spending time in the prison, he instead found them to be ‘specimens of turbulence, pugnacity and hardihood’, who were violent towards each other and members of the prison’s staff.^51^

Chesterton’s shock at the immoral conduct of these women is reflective of the broader difficulties lamented by officials when dealing with female prisoners. Within the reports of women ‘breaking out’, a deeper reading of what this actually entailed reveals that the women did not always act violently or destroy prison property. They were often punished for using bad or immoral language, shouting, expressing frustration at elements of the prison routine or simply breaking the rule of silence. These infractions were not exclusive to women’s prisons. Brown examined the complexity of Victorian prison organisation through the lens of the disturbances that took place within male public works prisons and found that, although large scale disorders did occur on rare occasions, small resistances were part of daily prison life.^52^ However, their categorisation as examples of ‘breaking out’ in the case of women was very much bound up in the broader expectations placed upon them to manage their tempers. Their refusal, or inability, to do so frequently resulted in them facing repeated periods in solitary confinement in dark cells. In this sense, it is possible to draw institutional parallels with the treatment of women in asylums. Showalter found that, between the 1850s and 1870, women were up to five times more likely than their male counterparts to be placed in padded cells for the use of immoral language.^53^

Infractions against the prison rules were answered by admonishments and the loss of marks earned through labour and good behaviour. With the approval of the medical officer, they were also met with reductions in diet, whether this be the loss of a meal or being placed on a diet of bread and water. Women were confined to punishment cells, sometimes for days at a time, or dark cells that were intended to let in little light or sound. However, prison authorities faced difficulties when attempting to restrain and punish incorrigible women due to the belief that continually depriving prisoners of food was more injurious to women than men and thus less sustainable. Guy advocated flogging women on medical grounds as he believed this ‘short bodily pain’ to be more merciful than repeated use of the bread and water diet.^54^ However, while flogging remained on the list of punishments for male convicts, although its infliction was increasingly regulated, it had been prohibited for women by an act passed in 1820.^55^ In 1857, the Deputy Superintendent of Millbank wrote to O’Brien to recommend the use of a gag to silence refractory women. However, O’Brien replied that such a practice was not recognised by law nor would it likely be ‘sanctioned by public opinion’.^56^

An issue raised by the Visiting Justices of Leeds Prison in April 1870, though occurring slightly after the period under examination here, was reflective of the long-term debates about controlling refractory women. They informed the Secretary of State that it had been necessary to erect some stocks in the prison to restrain several of the female prisoners. However, following his visit in May, Captain Powell, the Inspector of Prisons, directed against their use as he could find no authority for it. Interestingly, Powell referred to them as a form of punishment as opposed to restraint in objecting to their legality under the Prison Act 1865. A letter from the Secretary of State in June also declined to sanction their use.^57^ Historically, the stocks had been used as a punishment intended to shame those placed in them on a public stage. While the officials in Leeds considered them a vital means of restraining disobedient women, their use is also indicative of a desire to punish certain behaviours deemed inappropriate when perpetuated by women. This is further evidenced by the fact that there was no mention of the stocks being used for the male prisoners.

The issue of restraining women was not confined to debates over the use of the stocks in Leeds and instead it had troubled prison officials since at least the mid-nineteenth century. In January 1857, O’Brien received reports from the medical officers of Millbank and Brixton, each complaining about the difficulties of dealing with incorrigible women as repeated spells in the dark cells were found to cause a deterioration of health. In response, O’Brien visited the Middlesex County Lunatic Asylum at Colney Hatch and Hanwell Asylum to identify more effective means of restraining women. Following his visits, the stronger bedding used in the asylums was adopted to prevent the women from tearing it. The canvas dress, a coarse sack-like form of strait jacket intended to combat the women tearing off their clothes, was also adopted.^58^ The dress would be strapped over their convict dress and fastened by a belt and straps, but there were several instances where women removed the canvas dress using broken glass.^59^

By the mid-nineteenth century, the principle of non-restraint had been absorbed into the treatment of the insane in asylums unless restraint was believed to be for the safety of the patient.^60^ However, the use of the canvas dress as a means of restraint in prison was more complex as, in some cases, it was used in place of a punishment diet due to a belief that it was less dangerous to the prisoner’s health. In addition, instances of its use were listed within the tables of punishments. Although it was used only a handful of times in some years, and the strait jacket continued to be used on a greater scale, especially in Millbank, the adoption of the canvas dress shows some transference of practices from asylums. However, prison officials were largely not acknowledging that the women were mentally ill but instead were refractory and in need of containment, even if this was justified as being better for their health than other punitive methods.

A further measure adopted to suppress refractory women and prevent their contamination of other prisoners was the introduction of a penal class in Millbank in mid-1855. Its creation meant that incorrigible women who behaved with reckless abandonment in their constant breaking of the prison rules could be sent back from Brixton, Parkhurst and Fulham Refuge to Millbank. Although they accounted for a small proportion of the total prison population, the penal class proved the most difficult to manage. For example, in 1856, there were 31 women in the penal class at Millbank and 227 reports for misconduct. Comparatively, among the 511 women in the probation class, there were only 64 reports for misconduct.^61^ The Superintendent’s report for Brixton in the same year noted a significant improvement in the overall prison conduct due to the removal of the most incorrigible prisoners to Millbank.^62^ Wiener noted that, proportionally speaking, women were restrained more frequently than male convicts.^63^ However, this varied in different women’s prisons. The use of restraints, including strait jackets, handcuffs and the canvas dress was more numerous in Millbank, primarily for women in the penal class, than in other female prisons. In 1857, the 27 women in Millbank’s penal class were placed in handcuffs 62 times and in a strait jacket 110 times when compared to handcuffs being used only 20 times in Brixton as a whole.^64^ By the second half of the 1860s, the use of restraints in Brixton was rare, but they continued to be used between 100 and 200 times per year in Millbank despite repeated reports that the women were impervious to the punishments inflicted upon them.

Women were transferred back to Millbank to repeat the probation stage of discipline or to join the penal class for violent outbursts, repeatedly breaking the rules and for assaulting prison staff. However, in some cases, their removal was caused by relatively minor infractions such as the use of bad language and talking to their fellow prisoners, thus providing reinforcement to the argument here that certain behaviours, although not violent, were deemed less tolerable in women. Between 1855 and 1869, 254 women were moved from Brixton back to Millbank for misconduct. There were also 29 women moved back from Parkhurst Prison, which accommodated female convicts between 1863 and 1869. In addition, there were 134 women moved from Fulham Refuge back to Millbank for misconduct between 1856 and 1869. Their cases are particularly interesting as they had shown sufficiently good behaviour to earn their place in the refuge. In 1856, 14 women were removed following a disturbance caused by a misunderstanding over the remission of a portion of their original sentence. The Superintendent, Catherine Harpour, reported her belief that the women in question had not been in the previous stages of discipline long enough to prepare them for the diminished degree of control that was exercised in the refuge.^65^ This inability to manage their emotions without constant strict supervision was a recurring theme within reports of women disrupting the order of the prison, but it was also posited as an explanation for viewing cases of ill health, including those of insanity and of women attempting to harm themselves, with deep suspicion.

## Insanity, Suicide and the Infliction of Bodily Harm

In Victorian England, there were several public institutions intended to contain the women society placed within the brackets of ‘mad’, ‘bad’ or ‘sad’, including the prison, the workhouse and the asylum. Some women experienced incarceration in several of these institutions in the mid-nineteenth century. For example, a total of 70 women were removed from female convict prisons between 1853 and 1869 and placed in lunatic asylums.^66^ Analysing the various explanations given by the medical officers for their removal advances our understanding of the prison as a space in which the refractory and the insane were confined alongside each other and provides further evidence of the blurring of the line between their punitive, reformatory and medical treatment caused by the desire for prison regimes to exercise uniformity and deterrence.

The prison regime was believed to be the cause of insanity in a small number of cases. In 1853, a 12-year-old girl was removed from Millbank to a lunatic asylum due to the effects of having spent the greater part of her 8 months in the prison in separate confinement.^67^ In 1860, Rendle attributed the insanity of Brixton’s prisoner 1511 to her lengthy imprisonment. She had already served a 4-year sentence of penal servitude, and during her second spell behind bars, she began to show signs of insanity and was removed to an asylum.^68^ In other cases, the prison regime itself was not blamed for the onset of insanity, but it was believed that previously undetected unsoundness of mind had manifested due to the strains of incarceration.^69^ Again, this belief was not exclusive to female prisoners. Pentonville officials often emphasised prisoners’ pre-existing intrinsic weakness and incorrigibility to refute the criticism that the system itself had been the cause of their mental breakdown.^70^

Reports justifying the removal of some female prisoners to lunatic asylums include notable similarities with those discussing instances of ‘breaking out’. The reason given for the removal of three women from Millbank to Fisherton House Lunatic Asylum, for the criminally insane, in 1862 was their ‘extreme filthiness of conduct’ accompanied by ‘occasional outbursts of violence.’^71^ Similarly, three of the five women removed from Millbank in 1863 had been sent back from Brixton due to their poor behaviour, but it was suspected that they had been of unsound mind when they entered the prison.^72^ However, prison officials were concerned that women would intentionally act a certain way to facilitate their removal to an asylum. Griffiths referred to this as them ‘doing the barmy’.^73^ This scrutiny when deciding upon a prisoner’s state of mind meant that, during this period, 14 women were received back into convict prisons from lunatic asylums as their behaviour had improved. However, Rendle commented upon the danger of this, noting a case in 1864 in which he concluded that the removal of a women from Fisherton Asylum back to Brixton had triggered a second attack of insanity, and thus he recommended that she be transferred back to the asylum.^74^

The intense scrutiny of suspected insanity cases also extended to those of self-inflicted bodily harm and suicide attempts. Medical officers reported upon these cases and gave their opinion on appropriate treatments. Like instances of ‘breaking out’, these acts were often categorised as examples of poor behaviour as opposed to poor mental health. Journalist Frederick William Robinson published several pieces in the 1860s under the pseudonym of a prison matron. In his 1862 *Female Life in Prison*, he discussed women damaging themselves with scissors, fastening stay laces around their necks until respiration almost ceased and feigning madness to get into the infirmary.^75^ In 1856, a ‘troublesome woman’ died in Brixton after swallowing a needle. As she had previously confessed to swallowing broken glass, the coroner’s jury concluded that she had swallowed the needle accidentally.^76^ Similarly, in 1858, Superintendent Martin recorded that prisoner S. B. had incurred 24 misconduct reports for ‘constantly trying to injure herself’.^77^ The fact that these cases were not categorised, or treated, as suicide attempts nor even deliberate self-harm highlights not only the reticence of officials to acknowledge the potential for the prison regime to cause mental disorders but also the suspicion that women would go to great lengths of deception to subvert it.

Prisoner M. J. died in Brixton in September 1854 after throwing herself from the highest gallery of the prison’s west wing. One of two women to commit suicide in this period, Rendle stated she had been in good health since her reception in March and added that the only explanation for her actions was a ‘sudden attack of insanity’. However, his annual report demonstrated the inherent contradictions in defining mental ill health in prisons as he recorded two suicide attempts in the same year. He stated that one was by a woman of weak mind, but he nonetheless believed it to be a feigned attempt.^78^ Over a decade later, Rendle commented upon the rarity of suicide among female prisoners. Although he recalled the 1854 case, he reiterated his conviction that the woman in question had accidentally fallen when clandestinely attempting to get to the lower floor. He added that the few suicide attempts he had seen were by ‘badly conducted women’ who sought to cause disruption by ‘threatening to commit self-destruction’.^79^

Between 1853 and 1869, there were 45 recorded suicide attempts across the female convict prison estate.^80^ However, the true figure was almost certainly higher but unquantifiable due to the complexities of categorising certain acts as suicide attempts. For example, Superintendent Martin spoke of a ‘scheme’ in Brixton where women would feign suicide attempts to be placed in association. Instead, they were placed in refractory cells under restraint to prevent the infliction of any further self-harm.^81^ In only five of the cases were the women in question removed to an asylum. For many others, even when their actions were labelled as suicide attempts, medical officers stated that these acts were ‘probably feigned’ or were ‘evident cases of imposture’. When reporting upon the five suicide attempts in Millbank in 1859, Governor Bramly expressed his confidence that the women in question had the ulterior motive ‘of wishing to be considered of weak mind and therefore not amenable to the ordinary discipline of the prison and separate confinement’.^82^ Although these suspicions were not unique to women’s prisons and have been explored in relation to the management of the mental health of male convicts, the doubts about a prisoner’s intent were often rooted in gendered beliefs.^83^

When endeavouring to uncover the motivations behind suicide attempts and the commission of self-harm, prison officials pointed to explanations believed to be distinctly feminine. They reported that the women had not actually intended suicide and that the prison itself was not a direct cause of their actions. In 1856, a prisoner in Brixton attempted to hang herself after she had been reported for a ‘trifling offence’. While categorising the action as a suicide attempt, the Medical Officer pointed to the propensity for female prisoners to ‘frequently act on the impulse of the moment’ to explain her case.^84^ This again resonates with the broader rhetoric of the period, which posited female prisoners as being subject to impetuous outbreaks of extreme emotion. But, crucially, these outbursts were believed to be temporary and thus treated with short periods in isolation as opposed to in the prison infirmary.

Additional motivations given when women attempted suicide were concerns about their husbands and children. Communication was not only limited among those incarcerated but the regulation of contact extended also to a prisoner’s family beyond the prison walls. Although they were entitled to a limited number of letters and visits, many women in prison received neither, even when they had family on the outside. This isolation and uncertainty about the fate of their loved ones prompted violent conduct among some women and insanity in others. In June 1854, a woman described as a feeble invalid was transferred from Millbank to Brixton to be placed in the infirmary. She later received word of her daughter’s death and could get no news about her son. In April 1855, the Medical Officer detailed how she began having delusions that the prison was keeping her children from her and she claimed to hear them crying out. By June, she had become so violent that she had to be restrained. She was removed to Fisherton Asylum in July.^85^

A young woman with two children on the outside attempted to hang herself in Brixton in 1855 when she received news that her husband had been killed during the Battle of Sevastopol. The Medical Officer claimed she had not intended self-destruction but had acted due to being overwhelmed with ‘great mental distress and deep sorrow’. She was placed in the infirmary.^86^ Similarly, a woman in Parkhurst was placed in the infirmary in 1866 after she attempted to commit suicide whilst labouring under a ‘deep depression’ caused by the news that her husband had deserted their young children.^87^ These cases were likely treated with more sympathy as they stemmed from the archetypal feminine concern for husbands and children as opposed to impulses believed to display the more negative side of female nature. Their labouring under intense grief was used to conclude that they had not intended to take their own lives, and, crucially, their actions were not believed to have been motivated by a desire to somehow subvert or disrupt the prison regime.

## Debility, Disease and Physical Health

In 1865, Henry Roome, Parkhurst’s Medical Officer, observed that ‘when a large number of women are congregated together, under circumstances of a depressing character, in cells too small for continuous day and night occupation, and fed on food not very stimulating, it is not to be expected that they will be entirely free from diseases arising from debility’.^88^ Prison authorities were not only confronted with refractory, mentally ill and suicidal women, they also had to manage the physical health of those within their charge. Medical officers treated cases of ulcers, abscesses, diseases of the joints and bones, digestive complaints, kidney, lung, heart and brain diseases, epilepsy and respiratory issues, as well as venereal disease, menorrhagia (abnormally heavy menstrual bleeding), prolapses uteri and pregnancy. Many of these ailments were treated in a prisoner’s cell. However, in several cases, they required admission into the prison infirmary and some amelioration of the strict penal regime, whether this was an improvement in diet, the relaxation of labour requirements or being placed in greater association.

A problem faced by both male and female prisons in the nineteenth century was the condition of their inmates upon arrival. Those who populated these penal institutions overwhelmingly came from the most impoverished sections of society and many were in poor health when they entered the prison system. They were variously described as aged, debilitated or weak-minded and in need of immediate medical treatment. In 1860, Guy noted the high proportion of women in Millbank who had entered the prison as invalids and how, in that year alone, it had been necessary to move 23 of their number to Brixton’s infirmary.^89^ Despite the initial intention to send only healthy women to Parkhurst when it was made a women’s prison in 1863, in 1866, the Medical Officer commented upon the aged and debilitated condition of the women transferred there from Millbank.^90^

The quarterly returns made by convict prisons gave the names and ages of the people incarcerated and a brief note on the condition of their health.^91^ In Brixton, an average of 80 per cent of the women were deemed to be in ‘good’ health. However, the remaining 20 per cent were described as being in ‘delicate’ or ‘bad’ health, as being ‘feeble’, ‘not strong’, ‘weak-minded’, ‘insane’ or ‘invalids’. In the report of September 1860, 10 per cent of Brixton’s total population were described as invalids who required accommodation in the prison’s infirmary and a more substantial diet, and who could not perform certain labour tasks. However, consideration was given to incorporating them into Brixton’s regime as women of an advanced age, invalids and those with infants could still earn their progression through the disciplinary classes with good behaviour.

The offences that led women to the prison gates in this period predominantly resulted from a cyclical combination of alcohol abuse, offences related to prostitution and public disorder and poverty. In 1854, Brixton’s Chaplain claimed that 453 of the 664 women admitted during the year could trace the causes of their imprisonment to drink and keeping bad company.^92^ Similarly, in 1856, O’Brien consulted with the Medical Officer at Brixton and Miss Dyer, the Deputy Superintendent at Millbank, about the condition of the women in their prisons. Their reports acknowledged that some were deserving of pity due to the impoverished conditions from which they had come but almost half had either served previous prison sentences or were suspected to have been prostitutes and brothel keepers.^93^ These were factors believed to have contributed to their poor health upon reception into prison.^94^

Before they entered a convict prison, several women had served a number of shorter sentences in local prisons.^95^ There were repeated concerns raised by the medical officers that their health continually deteriorated with each spell behind bars. In 1855, Rendle treated 27 cases of debility and found that the prisoners’ health had begun to fail after 6 months as the prison caused feelings of weakness and loss of appetite. He argued that long sentences of penal servitude were more disadvantageous to the health of women as, although their previous domestic life meant they could bear the first 18 months of their sentences better than men, women remained confined behind the same prison walls for the duration of their sentence while male convicts were sent to public works prisons. Rendle stated his concern that sentences exceeding 3 years posed a distinct possibility of permanently damaging the prisoner’s health.^96^

An additional complaint made by Rendle in 1858 was that women who entered the prison in a poor state of physical health could not be placed on the punishment diet of bread and water if they broke the rules. In that year, there were 313 cases of women being placed in a refractory cell, but 207 had been kept on full rations. In addition, Rendle deemed 60 of the women received into Brixton to be invalids whose presence was problematic as they had to be accommodated in the infirmary at great expense. When adding that 11 of the 16 deaths in the prison that year had been women in very delicate health upon reception, he summed up a long-standing problem facing the criminal justice system, namely that they had ‘come to prison to die’.^97^ There were even cases where women had to be retained beyond the expiration of their sentence due to their poor physical state. Mary King Mitchell entered Brixton at the age of 16 years to commence a sentence of 5 years penal servitude in July 1854. Her sentence expired in July 1859, but she was too ill to be discharged and later died in the prison’s infirmary.^98^

Women were transferred between the different prisons that made up the female convict estate throughout the period under examination here. However, this transferal was not solely based upon their progression through the stages of discipline initially intended in 1853. In addition to the removal of refractory women back to Millbank, women were also moved between the prisons due to concerns about their health. In this period, male convict invalids could be moved to Dartmoor, Lewes and, later, Woking Prison, but there were no prisons especially designated for women in poor health. However, following its opening in 1853, Brixton increasingly received the women deemed unsuitable for the discipline in other prisons due to their health.

Between 1853 and 1869, there were 138 women moved to Brixton from either Millbank, Fulham Refuge or Parkhurst on medical grounds. This figure only includes cases where this reason for removal was made explicit, it does not include the refractory women who could not be removed back to Millbank due to concerns about their physical or mental state or the women retained in Brixton due to ill health despite their conduct warranting a progression to Fulham Refuge, as these figures were not routinely recorded. A reading of the quarterly reports delivered to the Home Office demonstrates that the women selected to finish their sentences in Fulham Refuge were generally in good health, not above the age of 40 years and were not repeat offenders. In addition, the infirmary at Fulham was only equipped to treat minor illnesses as it did not have a regular matron or nurses as the larger convict prisons did. Of the total 138 cases, 22 women had been removed from Millbank due to pregnancy. This was likely because, when it was rebuilt for women, Brixton included a nursery and four large rooms for convalescents and mothers with infants.^99^ Additional reasons given for the removal of women included consumption, scrofula and various pulmonary diseases. Debility and advanced age also accounted for a notable proportion and were issues repeatedly raised by Rendle as these prisoners could not be subject to the full rigours of ordinary prison life.

Brixton’s Superintendent frequently discussed the challenges posed by women who had been transferred there solely on medical grounds, without earning this progression.^100^ In 1857, she complained about wanting to send a group of refractory women back to Millbank to recommence their period of probation, but she was obliged to keep them on medical grounds.^101^ She further remarked in 1858 that there had been an increase in invalids and weak-minded prisoners who could not be subjected to strict discipline and, knowing this, they were deliberately defiant. Of 1,543 reports during the year, 395 were against prisoners not amenable to general punishment due to their physical or mental condition.^102^ Chaplain Moran also spoke of the disruption caused by women being sent to Brixton from Millbank before serving the requisite period in separation, thus losing part of the time reserved for their individual self-reflection.^103^

In 1867, Rendle successfully petitioned the Home Office for the early release of a girl of 17, who had been in the convict system since the age of 13 years. She was in the early stages of consumption, and he recommended that she be sent to her parents in the country for a change of air as he believed she would die imminently if kept in prison.^104^ But her case was rare, and many sick women remained behind the prison walls. Among their number were young women with consumption whose conduct was habitually bad, but their health meant they could not be punished or restrained. Rendle remarked that ‘the most troublesome prisoners … are young women who know that their ill-health will shield them from punishment, however bad their conduct’.^105^ Between 1854 and 1864, there had been 123 deaths, 51 of which were the result of consumption. A notable proportion of this number were young, refractory women. Rendle summed up the difficulty the prison faced when trying to contain but also care for these women when he stated that no one acquainted with their behaviour could venture any excuse for them, but the condition of their health required consideration.^106^

He took up the issue again in 1866 asserting that he was anxious to avoid seeming opposed to punishment but adding ‘faithfulness to duty compels me to state that there is far more serious danger to health and life from long periods, or from often repeated short periods of punishment diet, than a non-professional person can readily believe’.^107^ Though indicative of his own quandary, Rendle’s remarks served to further illustrate the difficulties posed by a medical officer’s dual loyalty to their prisoner patients and the prison service and the difficult task faced by the prison staff attempting to care for the health of their charges without impeding the institution’s disciplinary requirements. It was also a question that would play into the prison Directorate’s decision to rethink the arrangements in place for incarcerating the country’s female convicts in 1869.

## The Closure of Brixton

In 1864, the Directors of Convict Prisons acknowledged that none of the female establishments, except Fulham Refuge, had been specifically constructed for women. Although the building itself had been adapted, the system in Brixton was only a slightly modified version of that designed for men. They continued, ‘it is scarcely possible to expect that the best results of prison discipline can have been as yet attained in prisons of a makeshift character’. Therefore, they gained government approval to construct a new female convict prison at Woking.^108^ When reporting upon its progress in 1865, the Directors provided further justification for the closure of the other female convict establishments by stating that all due diligence had been taken to ensure the new prison addressed the ‘many deficiencies’ of the old system.^109^ This included additional spaces to provide more employment opportunities for women, such as mosaic tile work and shoe-closing, as well as a bakehouse, cookhouse and laundry. There would also be a designated penal ward, purpose-built officers’ dormitories and a bath house to ensure a high standard of cleanliness. In addition, the prison’s infirmary was built to cater for ‘all that can be desired for the reception and treatment of the sick, simple arrangements but cheerful looking and abundantly ventilated’.^110^

In December 1869, Brixton closed as an establishment for women and reopened in February 1870 as a light labour prison for men. From April 1869, Parkhurst, which had only accommodated women for 6 years, became a male convict prison for invalids. Their respective inmates were transferred to Woking, and henceforth, it would serve as England’s main female convict prison. Davie contextualised the growing frustration of officials in Brixton by the mid-1860s within a broader disillusion with the reformatory hopes of the previous decades, which prompted calls for greater severity in prisons.^111^ The push for uniformity in penal discipline and for it to act as a suitable deterrent from repeat offending was a key outcome of the select committee chaired by the Earl of Carnarvon in 1863 on the state of discipline in gaols and houses of correction—also known as the *Carnarvon Committee*.^112^ Wiener termed the ensuing legislation, including the Prison Acts of 1864 and 1865, as ‘an expression of the disciplinary subtext of Gladstonian Liberalism’.^113^

The closure of Brixton was bound up not only in wider considerations, notably the need to create more accommodation for male convicts, but also contemporary criticisms of the efficacy of the existing prison regimes. However, this study also provides reinforcement to Zedner’s conclusion that the closure was prompted by a specific dissatisfaction with attempts to imprison women.^114^ Within this, the experiences of the previous 16 years were crucial. Despite the intentions of officials in 1853, the system for female convicts required almost immediate modification. The question of balancing prisoners’ health needs with the disciplinary requirements of the institution, although continually raised, was never adequately addressed.

## Conclusion

In August 1855, Jebb professed the ambition that the different establishments in the female convict estate would be ‘components of the same system’, wherein the progression of female prisoners through the classifications would ‘work smoothly and well’.^115^ However, this system of orderly progression based upon the principles of reflection, discipline and, eventually, reform envisaged by Jebb was not achieved. Almost immediately after Brixton opened its gates, the system had to be modified and negotiated in practice. Prison officials, notably the medical officers, repeatedly raised concerns about the negative effects of prolonged imprisonment upon women and voiced frustration at the difficulties inherent in reconciling discipline and health.

By examining the previously underexplored transference of women between the different female convict establishments, this study revealed how this movement was often disrupted by considerations about health and behaviour. Despite the intention that they would be reclaimed and reformed as they progressed through the disciplinary stages, many women remained unmalleable to such efforts. In addition, although Brixton was initially intended to house those who had earned a place in the second and first classes, the prison increasingly received the women deemed too aged or debilitated for the full rigours of prison life. While prompting logistical difficulties, including their accommodation and care in the infirmary, the authorities also faced the problem of answering infractions of the prison rules when these women were deemed too weak to be punished.

The challenges of reconciling health and discipline prompted modifications and negotiations of the terms of their incarceration by the women themselves, as well as by those tasked with their custody. Instances of ‘breaking out’ offer just one insight into how prison officials attempted to understand and rationalise certain behaviours in women. The destruction of prison property and breaking the rule of silence was not confined to women’s prisons. However, the categorisation of these behaviours as examples of ‘breaking out’ was very much bound up in the broader expectations placed upon women but also upon prisons for women. Despite their fortress-like appearance posing the physical antithesis of the Victorian idea of a feminine space, prisons for women had a greater responsibility to morally reclaim their inmates and to provide punitive answers to displays of unfeminine behaviour.

Instances of ‘breaking out’, the destruction of prison property and the infliction of self-harm and suicide attempts were believed to have been predominantly prompted by poor behaviour as opposed to ill health and were often punished instead of medically treated. They provide examples of some women attempting to exercise agency over the conditions of their imprisonment by attempting to gain admission to the infirmary or containment in the dark cells where they could communicate with each other or, in Maria Copes’ case, movement to another prison. However, destroying prison property was also an outward display of the anxiety and uncertainty felt by those behind the cells doors who were facing lengthy periods in isolation.

The difficulties of incarcerating the healthy alongside the sick and the refractory with the insane proved to be a vital spoke on the problematic wheel of female incarceration. Although it turned again in 1869, powered by the renewed impetus on the part of the prison Directorate to rethink the arrangements in place for containing convicts of the gentler sex, the new female establishment at Woking did not, and perhaps could not, reconcile the question of caring for prisoner health whilst also adhering to obdurate penal regimes driven by the aims of uniformity and deterrence.

## Footnotes

1 *Parliamentary Papers* [hereafter *PP*] *Report of the Directors of Convict Prisons* [hereafter *RDCP*] 1856 (London: 1857), 338.

2 Seán McConville, *A History of English Prison Administration, vol. I*, *1750–1877* (London: Routledge & Kegan Paul, 1981), 425–28.

3 Lucia Zedner, ‘Wayward Sisters: The Prison for Women’, in Norval Morris and David J. Rothman, eds, *The Oxford History of the Prison: The Practice of Punishment in Western Society* (Oxford: Oxford University Press, 1995), 329–61, 331; Philip Priestley, *Victorian Prison Lives: English Prison Biography 1830–1914* (London: Pimlico, 1985), 69–70.

4 Neil Davie, ‘Business as Usual? Britain’s First Women’s Convict Prison, Brixton 1853–1869’, *Crimes and Misdemeanours*, 2010, 4, 37–52, 37.

5 Russell P. Dobash, R. Emerson Dobash and Sue Gutteridge, *The Imprisonment of Women* (Oxford: Basil Blackwell, 1986).

6 Lucia Zedner, *Women, Crime and Custody in Victorian England* (Oxford: Oxford University Press, 1991).

7 Martin J. Wiener, *Reconstructing the Criminal: Culture, Law, and Policy in England, 1830–1914* (Cambridge: Cambridge University Press, 1990), 122, 126.

8 Joe Sim, *Medical Power in Prisons: The Prison Medical Service in England 1774–1989* (Milton Keynes: Open University Press, 1990); Priestley, *Victorian Prison Lives*, 190.

9 For further details on the establishment of this disciplinary system, see *PP RDCP* 1853 (London: 1854), 305–10. For more detailed analyses of the marks system introduced to English convict prisons in 1864, wherein women could earn between six and eight marks a day to help them progress through the disciplinary classes, see Zedner, *Women, Crime and Custody*, 179–83; Davie, ‘Business as Usual?’, 41; Helen Johnston, *Crime in England 1815–1880: Experiencing the Criminal Justice System* (Abingdon: Routledge, 2015), 106–20.

10 Women were also incarcerated in many local prisons across England, usually for relatively short sentences. However, this study primarily focuses upon the management of women serving sentences of penal servitude in convict prisons to provide an exploration of the distinct challenges these establishments posed to prison officials. For more detailed analyses of the development of local prisons in nineteenth-century England, see Seán McConville, *English Local Prisons 1860–1900: Next Only to Death* (London: Routledge, 1995); Johnston, *Crime in England*, 88–105.

11 Stephen Watson, ‘Malingerers, the “Weak-minded” Criminal and the “Moral Imbecile”: How the English Prison Medical Officer Became an Expert in Mental Deficiency, 1880–1930’, in Michael Clark and Catherine Crawford, eds, *Legal Medicine in History* (Cambridge: Cambridge University Press, 1994), 223–41, 224.

12 Neil Davie, *Tracing the Criminal: The Rise of Scientific Criminology in Britain, 1860–1918* (Oxford: The Bardwell Press, 2005), 71.

13 *PP RDCP* 1854 (London: 1855), 392–93.

14 Hilary Marland, *Dangerous Motherhood: Insanity and Childbirth in Victorian Britain* (Basingstoke: Palgrave, 2004), 121–31.

15 Catherine Cox and Hilary Marland, ‘“He Must Die or Go Mad in This Place”: Prisoners, Insanity, and the Pentonville Model Prison Experiment, 1842–1852’, *Bulletin of the History of Medicine*, 2018, 92, 78–109, 81. See also Catherine Cox and Hilary Marland, ‘Broken Minds and Beaten Bodies: Cultures of Harm and the Management of Mental Illness in Mid– to Late Nineteenth-Century English and Irish Prisons’, *Social History of Medicine*, 2018, 31, 688–710.

16 *PP Report from the Select Committee on Prison Discipline Together with the Minutes of Evidence* (London: 1850), 123.

17 Hull History Centre TCGL 15 Gaol Committee Report, 1851.

18 *PP RDCP* 1853 (London: 1854), 308.

19 *PP RDCP* 1855 (London: 1856), 277.

20 The National Archives [hereafter TNA] PCOM 2/164/11 *Millbank Book of Questions and Suggestions 1855–1863*.

21 Henry Mayhew and John Binny, *The Criminal Prisons of London and Scenes of Prison Life* (London: Griffin, Bohn, and Company, 1862), 184.

22 Florence Elizabeth Chandler Maybrick, *Mrs Maybrick’s Own Story: My Fifteen Lost Years* (London: Funk and Wagnalls Company, 1905), 67, 86.

23 Susan Willis Fletcher, *Twelve Months in an English Prison* (New York: Charles T. Dillingham, 1884), 326.

24 *PP RDCP* 1854 (London: 1855), 392–93.

25 *PP RDCP* 1858 (London: 1859), 326.

26 For more on the public works prisons, see Alyson Brown, *English Society and the Prison: Time, Culture, and Politics in the Development of the Modern Prison, 1850–1920* (Suffolk: Boydell, 2003), Ch. 5.

27 *PP RDCP* 1854 (London: 1855), 389.

28 Arthur Griffiths, *Memorials of Millbank and Chapters in Prison History* (London: Chapman and Hall, 1884), 199, 208.

29 Cox and Marland, ‘“He Must Die or Go Mad in This Place”’, 99–100.

30 Watson, ‘Malingerers, the “Weak-minded” Criminal and the “Moral Imbecile”’, 223.

31 Davie, *Tracing the Criminal*, 70–75.

32 Jade Shepherd, ‘Feigning Insanity in Late-Victorian Britain’, *Prison Service Journal*, 2017, 232, 17–23, 19.

33 Dobash *et al.*, *The Imprisonment of Women*, 82.

34 Superintendent Emma Martin quoted in Mayhew and Binny, *Criminal Prisons of London*, 187.

35 Sim, *Medical Power in Prisons*, 39.

36 TNA PCOM 2/164/77-78 *Millbank Book of Questions and Suggestions 1855–1863*.

37 Griffiths, *Memorials of Millbank*, 200.

38 *PP RDCP* 1860 (London: 1861), 71.

39 TNA PCOM 2/164/125-129 *Millbank Book of Questions and Suggestions 1855–1863*.

40 Dr Winslow had previously written about Pentonville and declared that 14 per cent of the prison’s inmates were suffering from some form of mental illness. See Forbes Winslow, ‘Medical Society of London. Prison Discipline’, *The Lancet*, 29 March 1851, 357–60.

41 *PP RDCP* 1860 (London: 1861), 71.

42 TNA PCOM 2/164/125-129 *Millbank Book of Questions and Suggestions 1855–1863*.

43 *PP RDCP* 1860 (London: 1861), 71.

44 Cox and Marland, ‘Broken Minds and Beaten Bodies’, 690.

45 Mary Carpenter, *Our Convicts*, 2 vols (London: Longman, 1864), 214.

46 Leonore Davidoff and Catherine Hall, *Family Fortunes: Men and Women of the English Middle Class 1780–1850* (London: Hutchinson Education, 1987), 401, 450–51; Deborah Gorham, *The Victorian Girl and the Feminine Ideal*, volume 19 (Oxford: Routledge, 2014), see Ch. 6 for a discussion of how girls were taught to restrain their behaviour to prepare them for womanhood. See also Lydia Murdoch, *Daily Life of Victorian Women* (California: Greenwood, 2014) for more on how class, religion, age and race influenced how women negotiated society’s expectations in their daily lives.

47 Psychiatrists in the second half of the nineteenth century, notably Henry Maudsley, pointed to a correlation between increased irritability in women and their menstrual periods, which could lead to violent impulses. See Henry Maudsley, *Body and Mind; An Inquiry into their Connection and Mutual Influence, Especially in Reference to Mental Disorders* (New York: D. Appleton and Company, 1871), 78.

48 *PP RDCP* 1853 (London: 1854), 311. Elizabeth Fry was an early proponent of the belief that women required more feminine moral guidance. See Elizabeth Fry, *Observations on the Visiting, Superintendence and Government of Female Prisons* (London: John and Arthur Arch, 1827).

49 Zedner, *Women, Crime and Custody*, 209–13; Dobash *et al.*, *The Imprisonment of Women*, 84–88.

50 Bill Forsythe, ‘Women Prisoners and Women Penal Officials 1840–1921’, *British Journal of Criminology*, 1993, 33, 525–40, 527–28.

51 George Laval Chesterton, *Revelations of Prison Life with an Enquiry into Prison Discipline and Secondary Punishments*, 2 vols (London: Hurst and Blackett, 1856), 69–70.

52 Brown, *English Society and the Prison*, 4–5.

53 Elaine Showalter, *The Female Malady: Women, Madness and English Culture, 1830–1980* (London: Virago, 1987), 17, 81.

54 *PP Report of the Commissioners Appointed to Inquire into the Operation of the Acts (16 & 17 VICT. C.99 and 21 & 21 VICT. C. 3) Relating to Transportation and Penal Servitude* (London: 1863), 246.

55 Wiener, *Reconstructing the Criminal*, 94.

56 TNA PCOM 2/164/48-49 *Millbank Book of Questions and Suggestions 1855–1863*.

57 TNA HO 45/9685/A48397 Proposed use of stocks at Leeds Prison for punishing refractory female prisoners.

58 TNA PCOM 2/164/48-49 *Millbank Book of Questions and Suggestions 1855–1863*.

59 Mayhew and Binny, *Criminal Prisons of London*, 273.

60 Anna Shepherd, *Institutionalizing the Insane in Nineteenth-Century England* (London: Pickering & Chatto, 2014), 128; Andrew Scull, *The Most Solitary of Afflictions: Madness and Society in Britain 1700–1900* (London: Yale University Press, 1993), 100. See also Leslie Topp, ‘Single Rooms, Seclusion and the Non-Restraint Movement in British Asylums, 1838–1844’, *Social History of Medicine*, 2018, 31, 754–773. Topp highlighted how the parallels drawn between the use of solitary confinement in the prison system and the practice of seclusion—confining asylum patients in locked rooms—were refuted by those supporting seclusion as an alternative to mechanical restraint.

61 *PP RDCP* 1856 (London: 1857), 48.

62 *PP RDCP* 1856 (London: 1857), 322.

63 Wiener, *Reconstructing the Criminal*, 130.

64 *PP RDCP* 1857 (London: 1858), 59, 261.

65 *PP RDCP* 1856 (London: 1857), 354.

66 This figure was calculated by the author using the annual *Reports of the Directors of Convict Prisons* between 1853 and 1869.

67 *PP RDCP* 1853 (London: 1854), 87.

68 *PP RDCP* 1860 (London: 1861), 274.

69 *PP RDCP* 1862 (London: 1863), 273.

70 Cox and Marland, ‘“He Must Die or Go Mad in This Place”’, 100.

71 *PP RDCP* 1862 (London: 1863), 90.

72 *PP RDCP* 1863 (London: 1864), 76.

73 Griffiths, *Memorials of Millbank*, 208.

74 *PP RDCP* 1864 (London: 1865), 294.

75 Frederick William Robinson, *Female Life in Prison, by a Prison Matron*, 2 vols (London: Hurst and Blackett, 1862), 42.

76 *PP RDCP* 1856 (London: 1857), 342.

77 *PP RDCP* 1858 (London: 1859), 312.

78 *PP RDCP* 1854 (London: 1855), 392.

79 *PP RDCP* 1867 (London: 1868), 331.

80 This figure was calculated by the author using the annual *Reports of the Directors of Convict Prisons* between 1853 and 1869.

81 *PP RDCP* 1860 (London: 1861), 271.

82 *PP RDCP* 1859 (London: 1860), 43.

83 Cox and Marland, ‘“He Must Die or Go Mad in This Place”’; Shepherd, ‘Feigning Insanity in Late-Victorian Britain’.

84 *PP RDCP* 1856 (London: 1857), 342.

85 *PP RDCP* 1855 (London: 1856), 297.

86 *PP RDCP* 1855 (London: 1856), 298.

87 *PP RDCP* 1866 (London: 1867), 250.

88 *PP RDCP* 1865 (London: 1866), 254.

89 *PP RDCP* 1860 (London: 1861), 72.

90 TNA PCOM2/164/155 *Millbank Book of Questions and Suggestions 1855–1863*; *PP RDCP* 1860 (London: 1861), 250.

91 The author consulted the returns made in September of each year. Records for Brixton were included in the returns from December 1855, therefore the figures used in the current study cover the period from September 1856 to September 1869. See: HO 8/129; HO 8/133; HO 8/137; HO 8/141; HO 8/145; HO 8/149; HO 8/153; HO 8/157; HO 8/161; HO 8/165; HO 8/169; HO 8/173; HO 8/177; HO 8/181.

92 *PP RDCP* 1854 (London: 1855), 390.

93 *PP First Report from the Select Committee on Transportation together with the Minutes of Evidence and Appendix* (London: 1856), 68.

94 An extensive survey of the economic and social profiles of prostitutes in the Victorian period found that poverty was the principal cause for their entry into prostitution. See Judith R. Walkowitz, *Prostitution and Victorian Society: Women, Class and the State* (Cambridge: Cambridge University Press, 1980), 14–19.

95 For select case studies of women entering a constant cycle of reoffending, see Lucy Williams and Barry Godfrey, *Criminal Women 1850–1920: Researching the Lives of Britain’s Female Offenders* (Barnsley: Pen & Sword, 2018).

96 *PP RDCP* 1855 (London: 1856), 275.

97 *PP RDCP* 1858 (London: 1859), 326–27.

98 TNA HO 8/141/25 Quarterly Returns of Prisoners in Hulks and Convict Prisons, September 1859.

99 Note that there were still a few cases of childbirth occurring in Millbank when this study ends in 1869.

100 *PP RDCP* 1856 (London: 1857), 326.

101 *PP RDCP* 1857 (London: 1858), 258.

102 *PP RDCP* 1858 (London: 1859), 311.

103 *PP RDCP* 1856 (London: 1857), 333.

104 *PP RDCP* 1867 (London: 1868), 332.

105 *PP RDCP* 1859 (London: 1860), 273.

106 *PP RDCP* 1864 (London: 1865), 293.

107 *PP RDCP* 1866 (London: 1867), 260.

108 *PP RDCP* 1864 (London: 1865), 13.

109 *PP RDCP* 1865 (London: 1866), 10.

110 *PP RDCP* 1869 (London: 1870), 369.

111 Davie, ‘Business as Usual?’, 49.

112 *PP Report from the Select Committee of the House of Lords on the Present State of Discipline in Gaols and Houses of Correction* (London: 1863).

113 Wiener, *Reconstructing the Criminal*, 152.

114 Zedner, *Women, Crime and Custody*, 182.

115 TNA PCOM 2/164/1 *Millbank Book of Questions and Suggestions 1855–1863*.

## Notes

Rachel Bennett is a Research Fellow at the University of Warwick and works as part of the Wellcome Senior Investigator Award *Prisoners, Medical Care and Entitlement to Health in England and Ireland, 1850–2000.* Her research interests cover several aspects of crime and punishment in Britain and Ireland, with her most recent research exploring health and medical care in women’s prisons in England and Ireland since the mid-nineteenth century.

